# The effect of tenofovir in renal function in HIV-positive adult patients in the Roma health service area, Lesotho, southern Africa

**DOI:** 10.7448/IAS.17.4.19681

**Published:** 2014-11-02

**Authors:** Eltony Mugomeri, Dedré Olivier, Elmien van den Heever-Kriek

**Affiliations:** 1 Pharmacy, National University of Lesotho, Maseru, Lesotho; 2 Health Sciences, Central University of Technology, Free State, Bloemfontein, South Africa

## Abstract

**Introduction:**

The government of Lesotho introduced tenofovir disoproxil fumarate (TDF) for first-line antiretroviral treatment (ART), as recommended by the World Health Organization (1), in 2008. The use of TDF has been associated with renal toxicity (2); furthermore, renal function outcomes following the use of TDF has not been studied at Roma Health Service Area (RHSA) in Lesotho. Lesotho is a small landlocked country surrounded by South Africa. The study used an analytical design to compare retrospective creatinine clearance (CrCl) data of 312 (64%) antiretroviral treatment naïve adults exposed to TDF and 173 (36%) unexposed patients.

**Methods:**

Impaired renal function was defined as CrCl less than 50 mL/min calculated using the Cockcroft-Gault equation. The Ministry of Health and Social Welfare of Lesotho approved the study on 13 January 2012. The study included adult (excluding pregnant females) HIV patients enrolled on ART between December 2006 and December 2012 at St Joseph's Mission Hospital and at Nazareth Health Centre (RHSA). Patients at Nazareth Health Centre and at St Joseph's Mission Hospital made up 80% of the circa 4 116 HIV patients on ART. Only 485 patients met the set inclusion criteria.

**Results:**

In 56 patients (17.9%), TDF was found to be contraindicated. The use of TDF was marginally significant factor for renal toxicity (p=0.054) in univariate analysis, but was insignificant (p=0.122) in multivariate logistic analysis. Univariate (p<0.1) and multivariate logistic regression (p<0.05) were performed using STATA^®^ 11. Female gender (p=0.016), hypertension (p=0.009), and age>60 (p=0.004) were significantly associated with CrCl<50 mL/min outcome.

**Conclusions:**

In this study, TDF proofed to be a weak contributing factor of renal impairment. Routine baseline renal function screening should however be adopted to prevent patients with impaired renal function receiving TDF.
